# Maternal Exposure to Bisphenol-A and Fetal Growth Restriction: A Case-Referent Study

**DOI:** 10.3390/ijerph10127001

**Published:** 2013-12-11

**Authors:** Igor Burstyn, Jonathan W. Martin, Sanjay Beesoon, Fiona Bamforth, Qiaozhi Li, Yutaka Yasui, Nicola M. Cherry

**Affiliations:** 1 Division of Preventive Medicine, Department of Medicine, Faculty of Medicine and Dentistry, The University of Alberta, Edmonton, AB T6G 2G3, Canada; E-Mail: ncherry@ualberta.ca; 2 Department of Environmental and Occupational Health, School of Public Health, Drexel University, Philadelphia, PA 19102, USA; 3 Department of Laboratory Medicine and Pathology, Faculty of Medicine and Dentistry, The University of Alberta, Edmonton, AB T6G 2B7, Canada; E-Mails: jon.martin@ualberta.ca (J.W.M.); beesoon@ualberta.ca (S.B.); fiona.bamforth@albertahealthservices.ca (F.B.); 4 School of Public Health, The University of Alberta, Edmonton, AB T6G 1C9, Canada; E-Mails: qiaozhi@ualberta.ca (Q.L.); yutaka.yasui@ualberta.ca (Y.Y.)

**Keywords:** BPA, endocrine disruption, small for gestation age, birth weight, epidemiology

## Abstract

We conducted a case-referent study of the effect of exposure to bisphenol-A on fetal growth *in utero* in full-term, live-born singletons in Alberta, Canada. Newborns <10 percentile of expected weight for gestational age and sex were individually matched on sex, maternal smoking and maternal age to referents with weight appropriate to gestational age. Exposure of the fetus to bisphenol-A was estimated from maternal serum collected at 15–16 weeks of gestation. We pooled sera across subjects for exposure assessment, stratified on case-referent status and sex. Individual 1:1 matching was maintained in assembling 69 case and 69 referent pools created from 550 case-referent pairs. Matched pools had an equal number of aliquots from individual women. We used an analytical strategy conditioning on matched set and total pool-level values of covariates to estimate individual-level effects. Pools of cases and referents had identical geometric mean bisphenol-A concentrations (0.5 ng/mL) and similar geometric standard deviations (2.3–2.5). Mean difference in concentration between matched pools was 0 ng/mL, standard deviation: 1 ng/mL. Stratification by sex and control for confounding did not suggest bisphenol-A increased fetal growth restriction. Our analysis does not provide evidence to support the hypothesis that bisphenol-A contributes to fetal growth restriction in full-term singletons.

## 1. Introduction

Bisphenol-A is a high production volume chemical used as a building-block in the manufacture of plastics and resins. As a result, it is widely distributed in consumer products and in the human environment. Nearly all US residents are exposed to bisphenol-A to some extent according to the results of NHANES: 92.6% of the tested urine samples contained at least 0.4 µg/L of bisphenol-A urine [1]. In Canada as the whole, exposure is also widespread [2]. Research from the Canadian province of Alberta in 2005 on pooled sera of 26–30 year old pregnant women (in the first trimester) suggested that mean exposures were between 0.07 and 0.6 ng/g, with standard deviation of means of pools of 0.231 ng/g [3]. Health implications of exposure at these levels are currently hotly contested, with suggestive toxicological evidence and ambiguous or null epidemiological studies, leading to divergent regulatory regimes worldwide [4]. The need for high-quality epidemiology in resolving the uncertainty has been identified [5].

Animal experiments suggest that maternal exposure to bisphenol-A is associated with reduced birth weight in the offspring, but little similar human data on birth outcomes is available. Bisphenol-A exhibits estrogenic activity at very low concentrations, similar to levels of exposure in human populations [6,7,8], with the low-dose effects of particular concern according to some [8,9]. Fetal growth is one of the most readily available measures of neonatal well-being and is susceptible to other environmental insults (e.g., maternal smoking). Fetal growth restriction is a leading cause of perinatal morbidity and mortality [10,11] and is linked to poor growth in infancy [12] and neurobehavioral problems [13,14,15] in survivors. Based on *in vitro* studies and animal experiments, we hypothesize that estrogenic activity of bisphenol-A may affect fetal growth, perhaps differently in males and females (reviewed by Ranjit *et al*., [5]), a notion supported by one epidemiologic study [16]. Chou *et al*. [17] reported elevated odds ratio for small for gestational age in a sample of 56 male (but not female) newborns.

There are just a few epidemiological studies that shed light on the effect on bisphenol-A on pregnancy outcomes in humans and most of these were conducted in samples from general population. Lee *et al*. [16] reported a positive association between neonatal birth weight and maternal blood bisphenol-A measured at birth, with stronger effect in male infants. However, Padmanabhan *et al*. [18] observed no correlation between bisphenol-A in blood samples collected at delivery and birth outcomes, including birth weight. Similarly, Wolff *et al*. [19], measuring bisphenol-A in maternal urine in late pregnancy, did not find any associations with size or weight of 404 infants. In a case-referent study Philippat *et al*. [20], head circumference was found to increase (rather than decrease) with greater maternal urinary bisphenol-A during pregnancy; there was no evidence of effect modification by sex of infant. A prospective pregnancy cohort of 219 women in The Netherlands did not find an overall association between bisphenol-A in urine during pregnancy and fetal growth, but demonstrated a correlation in a sub-cohort of 80 mother-infant pairs with multiple exposure measurements [21]. The only study that examined influence of parental occupational exposures on birth weight reported that mothers with greater occupational exposure to bisphenol-A during pregnancy had offspring with lower birth weight [22]. Thus, conclusions from studies to date are inconsistent and there is still a need for well-designed and adequately powered studies of the influence of bisphenol-A on fetal growth.

Study of the human health impact of bisphenol-A has been hampered by the high cost of laboratory analyses of the compound: studies have been small, with limited power. A statistical design that *pools* biological samples across subjects minimizes analytical costs, while boosting the sensitivity of laboratory analytical methods (*i.e.*, reducing the problem of non-detectable concentrations in individual’s samples when exposure distribution lies close to ability of analytical instruments to quantify contaminants) and mitigating the impact of measurement error. Pooling entails combining equal-volume bio-specimens from individuals and analyzing the concentration of the contaminant of interest in the resulting combined specimen or pool. If pools are constructed separately for cases and referents then average differences in exposures between affected and healthy individuals can be examined and corresponding measures of risk calculated. The use of a pooling approach in exposure assessment [23,24,25] and epidemiology [26,27,28,29] is strongly supported. There is clear evidence that pooling biological specimens across subjects in exposure assessment can lead to studies that have identical power to those that conduct individual-level analyses at a fraction of a cost [26,27,28,29].

This study estimates bisphenol-A exposure from serum samples collected during pregnancy. It has been argued that no information can be obtained from blood or serum as bisphenol-A is rapidly cleared from blood and that any found in serum samples must result from contamination: urine sampling may be less subject to such effects [30]. We did not have access to urine samples. However, in a recent study of 50 paired human urine and serum, a strong correlation was found for those 20 subjects in whom bisphenol-A was identified in both blood and urine (*r* = 0.6, *p* < 0.01) [31]. Thus, we proceeded with this work using serum measurements of bisphenol-A, and with quality control measures to detect any contamination during collection and storage.

The research question addressed by this study is whether maternal exposure to bisphenol-A, as reflected in serum collected during pregnancy, is associated with *in utero* growth attained at delivery among full-term singletons. A supplementary question is whether such effects are confined to male infants.

## 2. Methods and Materials

### 2.1. Study Design

This was a case-referent study nested in a cohort of pregnant women, identified at 15–16 weeks gestation, in the Capital Health region, a previous administrative unit within the provincial universal healthcare infrastructure, in and around Edmonton, Alberta, Canada. The cohort was drawn from over 7,500 samples archived from women who elected to undergo a second trimester prenatal screen for Downs syndrome, open spina bifida, and trisomy 18. We are not aware of any studies on fluctuation of bisphenol-A in maternal sera throughout pregnancy but expect it to be at steady state till the time of delivery. Serum samples for these screens were collected between 15 December 2005 and 30 June 2007. The cohort was also restricted to women ≥18 years of age who gave birth to live singletons without evidence of malformation at birth, and who delivered at greater than or equal to 37 weeks of gestation. Data on birth outcomes were obtained from the Alberta Perinatal Health Program (APHP) by linkage on Personal Health Number, as in our previous work [32].

### 2.2. Case and Referent Definitions

Cases were defined as full term (≥37 weeks of gestation) singleton infants with birth weights below the 10th percentile, normalized for sex and gestational age [33,34]. Referents were defined as full-term singleton infants with birth weights between the 30th and 70th percentiles, normalized for sex and gestational age. Referents were individually matched (1:1) to cases on maternal smoking during pregnancy (yes/no), maternal age (±3 years) and infant’s sex. Maternal smoking during pregnancy was reported by the mother when interviewed at the time of delivery and recorded by APHP. We showed previously that this assessment method has nearly perfect specificity but sensitivity of only about 0.5 [35].

### 2.3. Exposure Assessment

Bisphenol-A concentrations were measured in pooled maternal sera. We used only one bio-specimen per woman. Individual 1:1 matching was maintained in the creation of pools, such that within a set (N = 7-9) of paired cases and referents, one pool contained all cases of the set, and the other pool contained all the associated referents. The minimum volume needed for bisphenol-A analysis was 2 mL, and to achieve this it was planned to pool aliquots from 8 to 9 individual serum samples of 250 µL each. When <250 µL was available for one of a pair, both case and referent were excluded, thus all pools contained between seven and nine individual samples. Serum samples had been archived at −80 °C and were defrosted in an upright position at room temperature. The defrosted serum samples were then mixed by gentle inversion for 5–10 s, and from each individual tube 250 µL of serum was pipetted into a new tube containing the pool. The pooled samples were placed into polyethylene bags at −80 °C before shipment for analysis.

### 2.4. Exposure Quantification

Total bisphenol-A concentrations in pooled sera were analyzed following the same general procedures adopted by the U.S. Centers for Disease Control and Prevention [36]. Briefly, 100 µL of each serum sample was fortified with internal standards (10 µL of 2 µg/mL mass-labelled bisphenol-A, 10 µL of a 2.5 µg/mL 4-methylumbelliferone glucuronide and mass-labelled 4-methylumbelliferone mix), and 50 µL of β-glucuronidase enzyme (*H. pomatia*, type H1) in ammonium acetate buffer (pH 5). The samples were mixed and incubated at 37 °C for 2 h to allow for any deglucuronidation, after which 80 µL of 1 M formic acid was added to terminate the enzymatic hydrolysis. The samples were made up to 1 mL volume with HPLC-grade water. The final sample extract was loaded onto a CTC PAL HTS autosampler (LEAP Technologies, Carrboro, NC, USA) and analyzed using a Thermo TSQ Vantage Mass Spectrometer with a Transcend Multiplexing System using in-line solid phase extraction and HPLC (Thermo Scientific, Mississauga, ON, Canada). The HPLC analytical column was a Thermo Hypersil Gold (50 × 2.1 mm, 1.9 µm particle size) and the SPE column was a Turboflow Cyclone-P SPE column (50 × 0.5 mm) (Fisher Scientific, Pittsburg, PA, USA). Injections of 100 µL were made, and elution was achieved using a gradient flow of methanol and water mobile phases. Detection was achieved by atmospheric pressure chemical ionization and tandem mass spectrometry (APCI-MS/MS). Quality control of the analysis was maintained by analyzing a method blank (calf serum), a duplicate serum sample, and a spiked calf serum sample along with every 20 real samples. The detection limit was 0.1 ng/mL, based on the lowest calibration standard which gave an instrumental signal to noise ratio of 3. For the statistical analysis all non-detectable measurements of bisphenol-A were replaced by 1/2 of the limit of detection.

### 2.5. Quality Control

To examine the potential for contamination of bisphenol-A during sample storage or pooling, quality control experiments were conducted. First, clean distilled water was placed in empty blood collection or storage tubes and were sent to the clinical laboratory for the same processing that real samples received. Second, pooling controls were prepared by pooling distilled water in the same manner as the real pooled serum samples.

### 2.6. Statistical Analysis

We used an analytical strategy, developed specifically for pooling, that estimated individual-level effects using associations between pool totals and odds of the outcome while conditioning on matched sets and total pool-level values of covariates [26]; details of the model are given in Equation (1), where *D* denotes case/control status (1/0) of *i*th pool among *K* pools formed in a study of *N* subjects, *U* continuous exposure of interest measured in a pooled specimen that take on value *u* for *i*th pool, *g* is the number of subjects in *i*th pool, *W* denotes pool specific sum of covariates that take on value *w* for *i*th pool, the parameter estimated are *α_i_ (pool-specific intercept) β*, and *γ*:

*logit* [Pr(*D* = 1∣*U* = *u*,*W* = *w*,*i*)] = *α_i_* + *β(u* × *g)* + *γw*,*i* = 1,..., *K* < *N* (1)

Both *β* and *γ* and their standard errors can be estimated by conditional logistic regression; they represent estimates of individual-level log odds ratios. Thus, the analysis used a conditional logistic regression model that allowed estimation of individual-level odds ratios (OR) and 95% confidence intervals (CI) per unit of exposure to bisphenol-A, after controlling for matching factors (maternal smoking (yes/no), maternal age, infant’s sex) and covariates and the number of aliquots in the pools. All analyses were controlled for, parity, maternal weight (≤45 kg *vs*. (>45 kg and <91 kg) *vs*. ≥91 kg), maternal height (<152 *vs*. ≥152 cm), and poor weight gain during the pregnancy. Separate analyses were carried out for pools for male and female neonates to examine the bisphenol-A effect in each sex. All statistical analyses were implemented in SAS9.3 (SAS Institute, Cary, NC, USA).

### 2.7. Ethics

Ethics approval for the project was granted by the Health Research Ethics Board of the University of Alberta.

## 3. Results

Serum from the 550 matched case-referent pairs were used to create 69 pools of cases (36 composed of males and 33 composed of females) and an equal number of pools of referents. All but six case pools and six referent pools had serum from eight women. Two case and control pools had serum from nine women and four pools had only seven. Quality control experiments did not indicate presence of contamination.

Table 1 indicates successful matching of cases and referents on maternal age and smoking during pregnancy. Mothers of cases had lower pre-pregnancy weight (2 *vs*. 1% ≤ 45 kg, and 5 *vs*. 8% ≥ 91 kg), were of shorter height (4 *vs*. 2% ≤ 152 cm), more likely exhibited poor weight gain in pregnancy than mothers of referents (3 *vs*. 2%), and more were nulliparous (46 *vs*. 33%). 

**Table 1:** Maternal characteristics of 550 cases and 550 referents (69 pairs of pools).

Characteristics	Case	Referent	*p*-value of χ^2^ test
Smoking any time during pregnancy			
(persons)	121	121	N/A
(number of pools)	(22)	(22)	(matching factor)
Age in years			
median	29	29	N/A
(inter-quartile range)	(25–33)	(25–33)	(matching factor)
Parity ^#^			
1	252 (46)	182 (33)	
2	141 (25)	171 (31)	0.01
3	78 (14)	89 (16)	
4–11	79 (15)	108 (20)	
Pre-pregnancy weight (≤45 kg) ^#^	12 (2)	4 (1)	0.1
Pre-pregnancy weight (≥91 kg) ^#^	25 (5)	45 (8)	0.09
Height (<152 cm) ^#^	23 (4)	11 (2)	0.04
Poor weight gain in pregnancy ^#^	19 (3)	12 (2)	0.1

Note: **^#^** count of persons (percentage).

**Figure 1:**
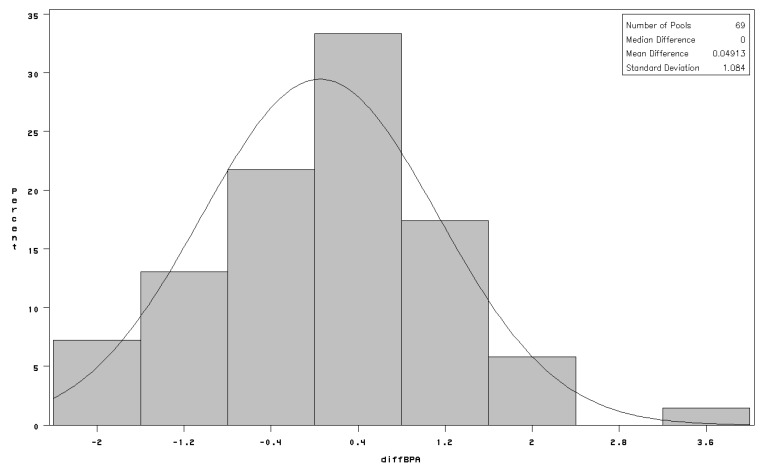
Distribution of paired differences between serum bisphenol-A concentrations (*diffBPA* in ng/mL) among matched pools of cases and referents; fitted line is normal distribution with estimated mean = 0 and estimated standard deviation = 1.

There was no appreciable difference in distribution of bisphenol-A concentrations in terms of pool geometric mean (GM = 0.5 ng/mL), geometric standard deviation (GSD) or the proportion—higher in cases—of pooled samples in which no bisphenol-A was detected (Table 2); the same pattern was observed when further stratifying on the sex of the newborn (details not shown). Mean difference (case-referent) in bisphenol-A concentrations between matched pools was 0.05 ng/mL with standard deviation of 1.08 ng/mL (Figure 1). There was no evidence that the odds of being born small for gestational age were related to exposure to bisphenol-A overall: all 95% CI were narrowly centered on 1.0 (Table 3). The results were very similar for boys and girls when analyzed separately (Table 3). Adjustment for potential confounders had no material impact on the results.

**Table 2:** Bisphenol-A measurements in 138 pools of maternal serum (ng/mL).

Case status (N)	Case (69)	Referent (69)
Number not detected	31	25
Minimum	<0.1	<0.1
Geometric Mean	0.49	0.50
Geometric Standard Deviation	2.47	2.29
Maximum	3.79	2.31

**Table 3:** Association of Bisphenol-A with small for gestation age at birth in live full-term singletons: continuous exposure metric (odds ratios per ng/mL bisphenol-A (OR) and 95% confidence intervals (CI)).

Model	Strata of neonates (n individual case-referent pairs)		
	All	Boys	Girls
	550	286	264
Unadjusted	1.01	1.02	0.99
	0.96, 1.07	0.95, 1.09	0.91, 1.09
Adjusted ^#^	0.96	1.01	0.97
	0.88, 1.05	0.91, 1.12	0.77, 1.21

Note: ^**#**^ Adjusted in conditional logistic regression for parity, maternal pre-pregnancy weight, maternal height (except for girls because there were too few “at risk” observations among referents to obtain stable estimates), poor weight gain in pregnancy; matched on child’s sex, maternal smoking and age.

## 4. Discussion

We observed no association between maternal serum concentrations of bisphenol-A in early pregnancy and being born small for gestational age in a sample of 550 growth-restricted infants and an equal number of referents. These effects were examined at exposure levels that are considered to be “low” (though not entirely atypical at median of 0.5 ng/mL in serum) [37] and thus are relevant to debate about risks of “low-level” effects of bisphenol-A in humans [8,9]. Because previous studies were conducted on individual samples, we cannot compare them to non-detects after pooling. The samples were in storage for several years before quantification of bisphenol-A. If there was degradation of bisphenol-A, samples of cases and referents would be equally affected so there is bias is not expected to arise. Our results strengthen the epidemiological evidence pertinent to human risk assessment for bisphenol-A, especially since they focus on a clinically relevant outcome (growth restriction in full term infants) rather than birth weight per se. Although the epidemiological literature on fetal growth and exposure to bisphenol-A is still limited, a coherent picture emerges when the totality of evidence is critically appraised.

Five clinic-based studies are relevant [16,17,18,19,20]. Three of these related bisphenol-A measured in blood at birth with fetal growth [16,17,18]. Lee *et al*. [16] investigated birth outcomes among 300 healthy pregnant women who delivered full-term infants at one antenatal clinic in South Korea; bisphenol-A in maternal serum and umbilical cord serum were quantified from blood samples collected at birth. They noted only one association: a higher (rather than lower) birth weight with greater BPA concentration. Chou *et al*. [17] studied birth outcomes in 97 maternal-neonate pairs with geometric mean of maternal levels of 2.5 ng/mL and geometric mean of umbilical cord levels at birth of 1.1 ng/mL. Association of small for gestation age in the fourth quartile of bisphenol-A concentration in maternal blood compared to the referent 1st quartile was reported among 56 boys. However, the effect was protective when 2nd and 3rd quartiles of exposure were compared to the reference group. The number of cases of small for gestational age was not reported but the total could not have been more than six based on the outcome definition given in the article [17] and consequently effect estimates are imprecise. Padmanabhan *et al*. [18] studied 40 women who delivered an infant at the University of Michigan Hospital in Ann Arbor during three months in 2006. When bisphenol-A in maternal blood collected at delivery was dichotomized at 5 ng/mL, there was no difference in either birth weight or length of gestation, although birth weight appeared to be marginally lower in those with bisphenol-A >5 ng/mL. Since effects on fetal growth occur during gestation and bisphenol-A has a short half-life, samples taken during pregnancy are more relevant than at term. Wolff *et al*. [19] addressed these limitations of earlier investigations [16,17,18] by investigating the association between urinary levels of bisphenol-A (inter-quartile range 0.7 to 2.3 ng/mL) in third trimester and size of singleton infant at birth among 367 primiparous women who were registered to deliver at Mount Sinai Medical Center in New York City (1998–2002). No association was found between urinary bisphenol-A and size (length, head circumference) gestational age or weight of the infant. Philippat *et al*. [20] conducted a case-referent study of male malformations nested in French mother-child cohorts recruited between 2002 and 2006 (72 cases and 215 referents). Bisphenol-A was quantified in maternal urinary samples collected between 24 and 30 weeks of gestational age in 48 cases and 143 controls from Nancy and Poitiers (France). The size (birth weight, length, head circumference) of the infant was extracted from hospital maternity records. The authors examined the association between bisphenol-A (and other exposures) and infant’s size, while employing a weighting scheme to correct for over-representation of malformations in the sample. Median urinary concentration of bisphenol-A was 3 ng/mL. A trend to increasing head circumference was reported (*p* < 0.01), with an estimate of 0.8 cm (95% CI: 0.2, 1.3) for bisphenol-A exposures ≥ 4.7 ng/mL *vs*. < 2.2 ng/mL: there was no harmful association reported. Thus, it appears that when exposures measurements are made in potentially etiologically relevant time-windows [19,20], the studies do not show an association of bisphenol-A with fetal growth.

The Generation-R study, a prospective population-based pregnancy cohort in The Netherlands, reported no significant effect of bisphenol-A in urine during pregnancy and fetal weight or fetal head circumference in the entire sample studied (219 women with at least one bisphenol-A measurement), but found indication of exposure-response association in a subgroup of 80 women with multiple measurements, where fetal weight appeared lower in the 2nd and 3rd quartiles of exposure [21]. There were weaknesses in this study both in the measurement of bisphenol-A (in two different laboratories which yielded rather different concentrations) and in uncertainties about the representativeness of women electing to provide three urine samples: it remains as the only population or clinic based study to report a significant effect in both sexes, even in a subgroup.

There is only one relevant occupational study: such studies have theoretical advantage over general population studies since they encompass high variability of exposures. Miao *et al*. [22] studied influence of paternal and maternal exposure to bisphenol-A exposure on the birth weight (obtained by maternal report) in 587 live-born singletons to persons enrolled in a cohort study of manufacturers of bisphenol-A and epoxy resin in China (2004–2008). The GM of maternal urinary bisphenol-A at the time of the study was 16.0, 2.0 and 0.6 µg/g creatinine in currently exposed mothers, spouses of exposed fathers and unexposed mothers, respectively. Exposure to bisphenol-A was determined through a combination of current personal air monitoring (inter-quartile range 1.5 to 16 µg/m^3^ 8-h time-weighted average) [38] and interview that elicited data on encounters with determinants of bisphenol-A exposure (e.g., specific jobs in bisphenol-A manufacture) that were combined to create a study-specific job-exposure matrix. The study reported decreased birth weight when both parents were exposed to bisphenol-A during the index pregnancy, with the effect greater in exposed mothers rather than fathers: average 168 g (*n* = 50) *vs*. average 91 g (*n* = 93) deficits. There was evidence of decreased birth weight in response to estimated exposure to bisphenol-A in the air at the time of the pregnancy. A relationship between workplace exposures and current urinary levels was reported but a correlation coefficient was not estimated. Birth weight was ascertained from parental recall, up to 15 years after the birth even in a censored analysis: 70% of index births occurred >5 years before interview. Any systematic error in recall with time would be confounded with time-trends in bisphenol-A exposure and weaken the study conclusions. Nevertheless, the suggestion of an effect of bisphenol-A on birth weight at exposure concentrations found in China >10 years ago cannot be discounted.

We determined that among higher quality epidemiological studies (in terms of power and appropriate timing of exposure measurements), two [19,20] show no association, while a third [21] found no effect overall but some decline in fetal weight in a subgroup, of uncertain provenance, with repeated exposure estimates. All three of these studies used urinary estimates of bisphenol-A. Our work was focused on the relevant outcome (fetal growth attained at birth) and measured exposures prior to birth in a robust matched case-referent design of 550 cases drawn from general population. It is particularly important to realize that, although our exposure assessment was conducted on pooled biological samples, the power of the case-referent analysis remained as high as if the exposure assessment was conducted at individual level [26,27]. Thus, our results are consistent with high-quality studies reported to date [19,20] and considered above to be methodologically sound. However, our work has limitations.

One of the limitations of pooling for exposure assessment is the loss of ability to look at heterogeneity of exposure within a pool and thereby fully describe the exposure distribution at the individual level. Such information would be invaluable for application of epidemiological results to risk assessment although it is not necessary for estimation individual-level odds ratios. If we were to repeat the study, it would have been advisable to also examine within-pool variability in exposure as recently recommended (by using a combination of pools and individual-level measurements), albeit at greater laboratory expense or reduction of the number of pools (and associated loss of power) [25].

All epidemiological studies of bisphenol-A suffer from poor understanding of etiologically relevant time window for exposure and the short biological half-life of bisphenol-A that potentially introduces “high” temporal variability in biologically effective dose. If sources of bisphenol-A are constant in the environment (as appears to be the case) then the short-half life may not prevent the establishment of a steady-state concentration in the body that is relatively insensitive to external exposure—the phenomenon of biological dampening [39,40]. Repeated exposure measurements during pregnancy would help address this concern as was done by others [21]. The pooling design in exposure assessment we utilized has a strength under these constraints because it mitigates problems with random measurement error but the systematic errors may still persist to bias the results [27]. Because measurement of bisphenol-A in the laboratory and construction of pools were blinded to case-referent status, measurement error in our study is expected to be non-differential. Although non-differential measurement error is expected to attenuate true exposure-response association, our results are so decidedly supporting the null that it is difficult to imagine they are the result of attenuated underlying positive association. In this context, it is important to note that measurement error typically does not alter the validity of the test of the null hypothesis (the calculated *p*-values are about the same as if there was not measurement error) [41,42]: our results are unambiguous in support of not rejecting the null hypothesis (*p* = 0.9).

We drew on cases and referents that were present within a select group of women who elected to undergo optional medical test. This group of women may not be representative of the general population but rates of small for gestational age, smoking, and other covariates do not appear to be atypical of the source cohort. It is also unlikely that selection for prenatal test for birth defect is related exposure to bisphenol-A, so that opportunity for confounding was minimized.

## 5. Conclusions

We conclude that our analysis does not provide evidence to support the hypothesis that bisphenol-A contributes to fetal growth restriction in full-term singletons at the time of birth of either sex at the studied concentrations. When considered in the context of prior epidemiological evidence, our results add support to the overall assessment that bisphenol-A does not have a measurable impact risk of being born small-for-gestational-age. The recent publication from The Netherlands [21] is provocative in that, using a rather sophisticated design and analysis relating fetal growth from ultrasound to concurrent bisphenol-A concentration, it appears to find effects not identified by other studies in a sub-group of the population. We agree with the authors of that publication that “we certainly need further evidence before we can conclude that in the general population bisphenol-A during pregnancy adversely affects fetal growth”. Indeed we would go further and concluded that, at present, the balance of evidence suggests that it does not.
